# How successful are oncologists in identifying patient distress, perceived social support, and need for psychosocial counselling?

**DOI:** 10.1054/bjoc.2000.1545

**Published:** 2001-01

**Authors:** W Söllner, A DeVries, E Steixner, P Lukas, G Sprinzl, G Rumpold, S Maislinger

**Affiliations:** Department of Medical Psychology and Psychotherapy, University Hospital, Sonnenburgstr. 9, Innsbruck, A-6020, Austria; Department of Radio-Oncology, University Hospital, Anichstr. 35, Innsbruck, A-6020, Austria; Department of ENT, University Hospital, Anichstr. 35, Innsbruck, A-6020, Austria

**Keywords:** cancer, psychological co-morbidity, oncologist estimation of patient distress, social support, psychotherapeutic support, social class

## Abstract

20–40% of cancer patients show emotional distress. Psychosocial support should be offered to severely distressed patients. However, little is known about the selection of patients to whom such support should be offered. This study investigated oncologists' ability to identify such patients. In a consecutive series of 298 cancer patients undergoing radiotherapy, distress, perceived social support and desire for supportive counselling were assessed using screening instruments. Simultaneously, 8 oncologists estimated patient distress and need for psychosocial support. A complete set of data was obtained in 80.2% of cases. Concordance of the oncologists' estimation of patient distress and perceived social support with the results of the screening instruments was weak (κ = 0.10 and κ = 0.05). Oncologists recognized the presence of severe distress only in 11 of the 30 severely distressed patients. Correct perception of distress was lower in patients with head and neck cancer and lung cancer and in lower class patients. Oncologists' recommendations for supportive counselling did not correlate with patient distress or the amount of perceived support but rather with progressive disease and less denial behaviour. Our results underline the need for educating oncologists in order to improve their ability to identify patient distress. © 2001 Cancer Research Campaign http://www.bjcancer.com


British Journal of Cancer (2001) 84(2), 179–185
© 2001 Cancer Research Campaign
doi: 10.1054/ bjoc.2000.1545, available online at http://www.idealibrary.com on IDEAL®

http://www.bjcancer.com

### How successful are oncologists in identifying patient distress, perceived social support, and need for psychosocial counselling?

**W Söllner[1], A DeVries[2], E Steixner[2], P Lukas[2], G Sprinzl[3], G Rumpold[1] and S Maislinger[1]**

[1]Department of Medical Psychology and Psychotherapy, University Hospital, Sonnenburgstr. 9, A-6020 Innsbruck, Austria; [2]Department of Radio-Oncology, University Hospital, Anichstr. 35, A-6020 Innsbruck, Austria; [3]Department of ENT, University Hospital, Anichstr. 35, A-6020 Innsbruck, Austria

**Summary** 20–40% of cancer patients show emotional distress. Psychosocial support should be offered to severely distressed patients. However, little is known about the selection of patients to whom such support should be offered. This study investigated oncologists' ability to identify such patients. In a consecutive series of 298 cancer patients undergoing radiotherapy, distress, perceived social support and desire for supportive counselling were assessed using screening instruments. Simultaneously, 8 oncologists estimated patient distress and need for psychosocial support. A complete set of data was obtained in 80.2% of cases. Concordance of the oncologists' estimation of patient distress and perceived social support with the results of the screening instruments was weak ($\kappa$ = 0.10 and $\kappa$ = 0.05). Oncologists recognized the presence of severe distress only in 11 of the 30 severely distressed patients. Correct perception of distress was lower in patients with head and neck cancer and lung cancer and in lower class patients. Oncologists' recommendations for supportive counselling did not correlate with patient distress or the amount of perceived support but rather with progressive disease and less denial behaviour. Our results underline the need for educating oncologists in order to improve their ability to identify patient distress. © 2001 Cancer Research Campaign http://www.bjcancer.com

**Keywords**: cancer; psychological co-morbidity; oncologist estimation of patient distress; social support; psychotherapeutic support; social class

Approximately 20–40% of cancer patients show emotional disorders requiring psychiatric or psychological intervention (Derogatis et al, 1983; Parle et al, 1996). In particular, cancer patients suffer from significant distress, tension, anxiety and feelings of helplessness especially before and at the beginning of radiotherapy (Munro and Potter, 1996; DeVries et al, 1998).

Oncologists play an important role not only in identifying psychological distress but also in preventing it by providing adequate information and basic emotional support to patients and their relatives. Additional psychosocial support should be offered to patients who are highly distressed and who receive poor support from their social network. For this purpose, the degree of distress and perceived social support should be assessed as early as possible (Kiss et al, 1995). Iscoe et al (1991) report that the assessment of patient distress shortly after the diagnosis of cancer is the best predictor for later adjustment disorder. The oncologists' estimation of whether and how severe a patient is distressed is often complicated by patients' denial. There are only a few studies which have investigated the skills of oncologists in identifying psychosocial problems. All of them found the oncologists' ability to detect psychological co-morbidity as unsatisfactory (Hardman et al, 1989; Sensky et al, 1989; Ford et al, 1994; Lambic et al, 1995; Newell et al, 1998; Passik et al, 1998). As a consequence, only 15–50% of cancer patients requiring psychiatric intervention are referred to mental health services for supportive counselling (Maguire et al, 1980; Hardman et al, 1989). To our knowledge, oncologists' awareness of the social support perceived by patients which might 'buffer' patient distress has not been investigated, so far.

*Received 19 June 2000*
*Revised 4 September 2000*
*Accepted 13 September 2000*

*Correspondence to:* W Söllner

The aims of the present study were (1) to assess oncologists' awareness of distress and social support of patients undergoing radiotherapy by comparing their assessment with the assessment by validated screening instruments, and (2) to identify factors that correlate with oncologists' success in identifying severely distressed patients in need of additional supportive counselling.

### MATERIALS AND METHODS

All consecutive cancer patients attending the Radio-oncology Unit of the Innsbruck University Hospital during a 6-month period from October 1997 to March 1998 were invited to participate in the study. Patients were informed about the aims of the study during the initial examination at the beginning of radiotherapy and were asked to fill in the following self-assessment questionnaires:

1. The German version of the Hospital Anxiety and Depression Scale (HADS, Zigmond and Snaith, 1983; Herrmann, 1997), a valid and reliable 14-item instrument to assess anxiety and depression in physically ill subjects. Several studies show good sensitivity and specificity of the HADS for detecting psychiatric co-morbidity (Hopwood et al, 1991; Herrmann, 1997; Hoffman et al, 1998). For use in cancer patients, Razavi et al (1990) recommended the use of the total score to assess the general amount of psychological distress. They developed cut-off values to differentiate between mild (HADS total score <13), moderate (13–18) and severe (>18) distress.
2. The Hornheide Questionnaire, Short Form (HQ, Strittmatter 1997) is a 9-item screening instrument, specially developed to assess psychosocial problems of cancer patients. Patients rate symptoms of distress and amount of perceived social support on ordinal scales (values 0–5). Validity and reliability have been proved to be good (Rumpold et al, 2001). The authors of the instrument developed cut-off points for each dimension of distress and for the total score in order to identify patients with high distress requiring psychosocial support. These patients are further classified into 'moderately' and 'severely' distressed subjects, the latter experiencing distress in more than one dimension and exceeding the cut-off value of the HQ total-score.

3. The Questionnaire to Assess the Need for Psychosocial Support (Söllner et al, 1998) is a short questionnaire with yes/no questions for assessing social and financial problems, perceived support from persons of the social network, and interest in supportive counselling by a psychotherapist and/or a social worker. Comprehensibility, content validity, and reliability are satisfactory.
4. In order to assess two important intervening variables, namely patients' tendency to minimize problems and their compliance with oncological treatment, we used the 'Minimizing problems' and the 'Compliance' subscales of the Freiburg Questionnaire for Coping with Illness (Muthny, 1989), a validated, 35-item self-assessment questionnaire widely used in German-speaking countries.

We collected clinical data by using chart-notes, and sociodemographic data of the patients by using a short form filled-in by the patients. Social class was assessed by the method of Kleining and Moore (1968) which is often used in Central Europe. Patients were assigned to a social class on the basis of their current or last occupation and employment status. Examples of different categories of social class are given in Table 1. Respondents who could not be classified by current or last occupation were assigned to a social class on the basis of the occupation of the head of the household.

To determine how oncologists estimate patient distress and available social support as well as their need for additional professional psychosocial support, we developed a short questionnaire (Oncologist Questionnaire, OQ) on the basis of the questionnaire of Hardman and co-workers (1989). The radio-oncologists completed the OQ at the same time the patients filled in their questionnaires immediately after the medical interview at the beginning of radiotherapy. To compare the radio-oncologists' assessment with that of other oncologists, we asked ENT surgeons in charge of the head and neck cancer patients of our study sample to fill in the OQ, too. They answered the OQ after conducting a medical interview with the patients at the beginning of the hospital stay (usually one day prior to the assessment by the radio-oncologists).

Oncologists assessed the following dimensions:

1. Patient distress (categories: mild – moderate – severe).
2. Perceived support from the social network (categories: good – moderate – poor).
3. Need for psychotherapeutic support (categories: no need – less urgent need – urgent need).
4. Need for counselling by a social worker (categories: no need – less urgent need – urgent need).

The prevalence of patient distress, perceived social support and need for supportive counselling were calculated from both the patients' and the oncologists' reports. Concordance between the patients' and the oncologists' assessment was measured using Kappa statistics. Kappa = 1 means full agreement, Kappa = 0 means that agreement can be explained solely in terms of chance, and Kappa < 0 is found if the observers disagree. The following statistical measures were calculated as indicators of the oncologists' awareness of patient distress and social support: sensitivity, specificity, and overall disagreement level (false positive and false negative rates).

Predicting variables of the oncologists' performance included sociodemographic and medical characteristics of the patients, patient compliance with standard treatment, and a coping style characterized by denial. These variables were analysed in two steps: in a first step, we performed univariate analyses using $\chi^2$ tests or univariate ANOVA, and in a second step, logistic regression analyses (Wald method). All statistical comparisons were two-sided, using $P < 0.05$ as the level of significance. All computations were done with the help of the Statistical Package for Social Sciences (SPSS9.0).

### RESULTS

Out of 306 consecutive patients 298 were eligible for inclusion in the study (8 patients were excluded because of very poor physical condition), and 254 of them (85.2%) participated in the study. 11 patients refused to participate, 6 patients could not fill in the questionnaires because of technical problems (like missing spectacles), and in 27 cases the questionnaires were not handed out because of organizational problems. Non-participants did not differ from participants in any of the collected sociodemographic and clinical data. Characteristics of the patients participating in the study are shown in Table 1.

Patients were examined by 8 radio-oncologists, 3 female and 5 male, between 34 and 43 years of age. Two of them were senior radio-oncologists and 6 were experienced registrars with an advanced level of professional education (3–11 years of postgraduate medical experience; 3–5 years in radio-oncology). 7 radio-oncologists were from upper middle class families and one radio-oncologist grew up in a lower class milieu. 6 ENT surgeons (5 male and one female, between 32 and 40 years of age) participated in the study. 3 were senior ENT surgeons, and 3 were registrars with 3–6 years experience in the ENT department. None of the oncologists had received any formal training in psychosocial assessment or communication skills. Radio-oncologists completed the OQ in 280 out of the 298 cases (94.0%). ENT surgeons filled-in the OQ in 49 out of the 53 head and neck cancer patients (92.5%). A complete set of patient and radio-oncologist questionnaires appropriately filled in was obtained in 239 cases (80.2%).

### Oncologists' awareness of patient distress and perceived social support

Patients' evaluation of distress on the HADS showed that 61.4% had mild, 25.1%, moderate and 13.5% severe distress. Using the HQ, a slightly higher prevalence of moderate (31.5%) and severe (16.6%) distress was obtained. On the other hand, oncologists classified 27.8% of the patients as mildly, 56.5% as moderately, and 15.7% as severely distressed. Kappa statistics showed only poor concordance between the oncologists' estimation and the patients' reports ($\kappa = 0.10$ with the HADS and $\kappa = 0.11$ with the HQ). Oncologists tended to judge patient distress to be higher than patients themselves; overall disagreement level with the HADS

© 2001 Cancer Research Campaign

**Table 1** Sociodemographic and clinical characteristics of the patients[1]

| | % (*n* = 254) |
|---|---|
| Age (years) | |
| <50 | 25.2 |
| 50–59 | 31.1 |
| 60–69 | 28.0 |
| >70 | 15.8 |
| Gender | |
| Male | 44.5 |
| Female | 55.5 |
| Marital status | |
| Married | 65.0 |
| Single | 12.6 |
| Divorced/separated/widowed | 19.2 |
| Living situation | |
| Living alone | 13.0 |
| Living with a partner | 71.7 |
| Living with family of origin | 5.1 |
| Education | |
| Elementary school | 39.4 |
| Secondary school | 33.9 |
| College/University | 20.5 |
| Socio-economic status[2] | |
| Upper class/upper middle class | 13.4 |
| Lower middle class | 31.7 |
| Upper strata of the lower class | 42.7 |
| Lower strata of the lower class | 12.2 |
| Type of cancer | |
| Breast cancer | 26.4 |
| Head and neck cancer | 20.9 |
| Lung cancer | 11.4 |
| Other | 41.3 |
| Stage of cancer | |
| I or II | 41.7 |
| III or IV | 49.2 |
| No classification possible/unclear | 9.1 |
| Metastasis | |
| No | 68.1 |
| Yes | 14.2 |
| No classification possible/unclear | 17.7 |
| Cancer recurrence | |
| No recurrence | 73.6 |
| Recurrent cancer | 22.0 |

[1]Because of missing data percentages do not always add to 100.
[2]Sufficient data to reliably estimate the social class of the patients were available in 164 cases only. Examples of upper class/upper middle class: academics, executive managers; lower middle class: white collar workers without leading position; upper strata of the lower class: skilled workers; lower strata of the lower class: unskilled workers.

was 59.5% (false positive rate = 46.1%, false negative rate = 13.4%) and with the HQ 57.5% (false positive rate = 37.6%, false negative rate = 19.9%). Using the lower cut-off values of the HADS, to detect moderate or severe distress, oncologists identified 69 out of 86 'cases' (sensitivity = 80.2%, specificity = 32.8%). However, when the higher cut-off values to detect only severe distress is employed, sensitivity of the oncologists' estimation of distress decreased to 36.7% (oncologists noticed high distress only in 11 of the 30 severely distressed patients), while specificity increased to 87.6% (Table 2).

There was considerable difference between the oncologists in their ability to identify patients with significant distress ($\kappa$-values ranging from –0.05 to 0.26). However, none of the oncologists reached a Kappa-value representing at least a moderate concordance ($\kappa > 0.30$). Radio-oncologists did not differ from surgeons in the identification of patient distress (both groups showing $\kappa$-values of 0.10 as compared with the HADS).

The majority of patients reported receiving good support from family members and peers, whereas 21.7% of the patients perceived only moderate or poor support. Concordance of the oncologists' estimation of available support with the patients' perception of support was poor ($\kappa = 0.05$). Sensitivity and specificity of oncologists' estimation of social support are shown in Table 2.

### Oncologists' awareness of the need for psychosocial counselling

At the Department of Radio-Oncology a psychotherapeutic consultation-liaison service as well as the services of a social worker had been available for several years. Both services are well integrated in the system of care delivery to oncology patients, the psychotherapist and the social worker being regularly present at the department for some hours during the week. Previous studies (Worden and Weisman, 1980; Söllner et al, 1999) showed that patients' interest in psychosocial support is influenced by what coping strategies they employ in dealing with cancer (e.g. denial). We therefore compared the oncologists' recommendations for supportive counselling not simply with patients' desire for such support but with an algorithm proposed by the International Consensus Meeting on Psychosocial Support in Cancer Patients organized by the Swiss Cancer League (Kiss et al, 1995). The consensus statement proposed that psychotherapeutic support should be offered if one of the following conditions is met: (1) high levels of patient distress, (2) poor social support, and (3) patients' desire for such support. With regard to counselling by a social worker, we developed a similar algorithm: such counselling should be provided if (1) patients suffer significant financial or occupational problems or (2) desire such counselling themselves.

Oncologists recommended psychotherapeutic support urgently in 8.8% of patients, and less urgently in 67.0%, and counselling by a social worker urgently in 3.2%, and less urgently in 35.1% of patients. Concordance between the psycho-oncological algorithm and the oncologists' recommendation for psychotherapeutic support was low ($\kappa = 0.08$). Though concordance of oncologists' recommendation of counselling by a social worker with patient assessment of their social problems was higher than for psychotherapeutic support ($\kappa = 0.22$), counselling by a social worker was not recommended in 42% of patients with severe social problems (Table 2).

### Predictors of concordance between oncologists' and patients' evaluation

We performed logistic regression analyses with concordance/discordance of perception of (a) distress, (b) perceived social support, (c) need for psychotherapeutic support, and (d) need for counselling by a social worker as the dependent variables. Patient characteristics (sociodemographic data, disease characteristics, patients' tendency to minimize problems and compliance with cancer treatment) that showed correlations with the dependent variables in univariate analyses were entered first as independent

© 2001 Cancer Research Campaign

**Table 2** Oncologists' awareness of elevated distress, diminished social support, and need for supportive counselling in cancer patients ($n$ = 239)

| Outcome measures | Missing data[1/2] | Patient screening[3] or consensus[4] % | Oncologists' perceptions % | Sensitivity % | Specificity % | Disagreement level % (false –/ false +) |
|---|---|---|---|---|---|---|
| Distress | 12/4 | | | | | |
| Moderate or severe | | 38.3 | 72.3 | 80.2 | 32.8 | 48.9 (7.6/41.3) |
| Severe | | 13.2 | 16.2 | 36.7 | 87.6 | 19.3 (8.5/10.8) |
| Support perceived from social network | 11/11 | | | | | |
| Moderate or poor | | 23.2 | 26.3 | 27.7 | 74.7 | 35.5 (15.7/19.8) |
| Poor | | 10.1 | 5.7 | 14.3 | 94.9 | 12.9 (8.3/4.6) |
| Need for psychotherapeutic support | 9/4 | | | | | |
| Less urgent | | 41.4 | 74.9 | 80.6 | 27.9 | 49.4 (8.4/41.0) |
| Urgent | | 20.0 | 8.9 | 26.7 | 95.6 | 18.0 (14.5/3.5) |
| Need for counselling by a social worker | 14/4 | | | | | |
| Less urgent | | 45.3 | 39.1 | 53.5 | 74.0 | 35.1 (20.7/14.4) |
| Urgent | | 14.7 | 3.0 | 12.9 | 98.4 | 13.6 (12.2/1.4) |

[1]Missing data from patients' questionnaire. [2]'Don't know' answers from OQ. [3]Screening instruments: HADS for distress and HQ for social support. [4]Consensus Conference on Psychosocial Support in Cancer Patients (Kiss et al, 1995) for the estimation of the need for psychotherapeutic support and counselling by a social worker.

variables. In the next step, characteristics of the oncologists (age, sex, years of professional education, senior oncologists vs. registrars) were included.

Oncologists more often failed to correctly identify distress in head and neck cancer (67%) and lung cancer (68%) compared to other cancer patients (52%) and in patients belonging to the lowest social class (78.9%) compared with the middle class and the upper strata of the lower class patients (54.0%). Employing logistic regression analysis 71.8% of the cases (correct identification of distress) could be predicted by these 3 variables: head and neck cancer (R = 0.23; $P$ = 0.001; OR = 2.6; 95% CI = 1.5–4.8), lung cancer (R = 0.10; $P$ = 0.099; OR = 1.8; 95% CI = 1.0–3.5) and lowest social class (R = 0.13; $P$ = 0.033; OR = 2.4, 95% CI = 1.1–5.3). Correct classification of distress did not correlate with patients' tendency to minimize problems, nor with their compliance with treatment.

The oncologists' estimation of perceived support from the patients' social network showed significant correlation with patients' marital status. Correct perception of social support was higher in married patients than in single, divorced or widowed patients (67.6% vs. 51.4%). However, perception of social support did not correlate with real life situations namely, living alone or together with a partner or family. Again, patients' social class was a predictor of correct perception: oncologists failed to correctly identify perceived social support more often in patients of the lowest class (concordance rate 39.9% vs. 67.2% in higher classes). Using regression analysis these two variables predicted correct perception of social support in 68.6% of cases (marital status: R = 0.12; $P$ = 0.048; OR = 1.6; 95% CI = 1.0–3.4; lowest social class: R = 0.16; $P$ = 0.018; OR = 2.3; 95% CI = 1.2–4.7).

Patients whose need for supportive counselling by a psychotherapist was missed by the oncologists suffered more often from progressive disease and exhibited more denial behaviour than patients whose needs were recognized correctly or were overestimated. Applying regression analysis, only progressive cancer was identified as predictor variable for correct perception of the need for psychotherapeutic support (R = 0.09; $P$ = 0.074; OR = 1.5; 95% CI = 1.0–2.4).

Oncologists' perception of whether patients should receive counselling by a social worker correlated with cancer diagnosis: there was underestimation in head and neck cancer and overestimation in lung cancer patients. Correct estimation was higher in patients with low denial behaviour. Applying regression analysis, concordance in recommending support by a social worker was correctly predicted in 73% of cases by three of the patients' characteristics: low denial (R = –0.19; $P$ = 0.008; OR = 0.5; 95% CI = 0.3–0.8), lower education (R = –0.10; $P$ = 0.075; OR = 0.6, 95% CI = 0.4–0.9), and rural residence (R = 0.18, $P$ = 0.012; OR = 1.9; 95% CI = 1.1–3.2).

Oncologists' characteristics such as age, gender, and years of professional education did not influence the concordance of the oncologists' and the patients' perceptions of any of the outcome variables.

### DISCUSSION

In a cross-sectional study we investigated the oncologists' ability to identify cancer patients who suffer significant distress, perceive poor social support and are in need of psychosocial counselling. We obtained a high recruitment rate of patients and oncologists.

Whereas the oncologists' sensitivity in recognizing moderate distress was relatively high, their ability to detect severe distress was low. In agreement with the findings of Hardman et al (1989), Lambic et al (1995), and Newell et al (1998), the better recognition of moderate distress was only achieved at the cost of a high false-positive rate. This might be a consequence of the oncologists' tendency to give an intermediate rating of distress ('moderate' instead of 'mild' or 'severe') which could have been the result of uncertainty in the estimation of patient distress. Sensky and associates (1989) suggested that physicians might accurately recognize emotional distress or affective disorder, in a proportion of patients, but might not acknowledge them as serious disturbances or 'psychiatric cases'. We cannot exclude that such missing acknowledgement of distress plays a role in our study, although this seems to be less the case where physicians rate the presence and amount of cancer-related distress, as in our study, instead of rating 'psychiatric cases' of anxiety and depression.

Passik and colleagues (1998), in a study of the identification of patient depression by oncologists, found inaccurate detection of

 © 2001 Cancer Research Campaign depression more often in patients with poor performance of activities of daily living and in patients suffering from cancer pain. They could not identify any associations with sociodemographic characteristics of the patients (but they did not assess patients' social class), and they did not report any associations with the localization of cancer. In our study, however, distress was more often inaccurately recognized in patients with head and neck cancer, in patients with lung cancer, and in patients belonging to the lowest social class. Why this is so is at present a matter of speculation. Discussions with the oncologists, when confronted with the results of this study, support the assumption that communication barriers due to impaired speech in patients with surgery for head and neck cancer might have influenced their awareness of patient distress. Moreover, most of the radio-oncologists are from upper middle class origin, and might be less familiar with the expression of psychological distress in lower class patients.

Concordance between the oncologists' and the patients' perception of the emotional and practical support received by the patients from their social network was even lower than for distress. In estimating the amount of support, the oncologists seem to rely more on formal criteria (patient's marital status) than on the patient's real living situation (living alone, with a partner or with his/her family). Again, the oncologists' perception of perceived social support was associated with patients' social class. Concordance was lower in patients from the lowest social class. Physicians judged these patients as receiving less support than the patients did themselves. In a recent American study, VanRyn and Burke (2000) found similar results and argued that lower social class is frequently associated with stereotyping.

The oncologists tended to overestimate the need for psychotherapeutic support in comparison to the recommendations of the psycho-oncologic consensus conference. On the contrary, they underestimated the need for counselling by a social worker. However, psychotherapeutic support was urgently recommended in 27% of the severely distressed patients only. The poorer agreement of the oncologists' perception with the recommendation of the psycho-oncologic Consensus Conference in patients with progressive cancer might be explained by higher denial behaviour in these patients. Counselling by a social worker was recommended only in half of the patients with significant social problems. Higher agreement between oncologists' recommendation and the guidelines of a professional consensus meeting in less educated patients from rural areas may be the consequence of more obvious difficulties of the social situation in these subjects.

Some of the possible reasons for poor concordance between the oncologists' and the patients' perceptions are considered below:

1. Identification of severe distress by the oncologists might have been hampered by patients' denial behaviour. However, in our study concordance of the oncologists' and the patients' perception of distress was not associated with patient coping by denial. Whilst denial did not seem to influence physicians' recognition of distress, it appeared to influence whether or not patients were referred for psychosocial support. When confronted with these findings, the radio-oncologists who participated in the study argued that they had been aware of this but had felt that it would have been inappropriate to refer patients with obvious denial behaviour to a psychotherapist or a social worker.
2. Low concordance between oncologists' and patients' perceptions might also be due to the time factor. Radio-oncological wards are characterized by high out-patient turnover, high technical demands and a heavy work-load that might hinder identification of severe distress or poor support in patients. However, in our study adequate time (about one hour) was available for the medical interview with the patients. Furthermore, head and neck surgeons whose working conditions differ from those of radio-oncologists (high workload as well, but easier access to conversation with in-patients) showed as poor a concordance with patient perceptions as radio-oncologists.
3. Previous studies support the assumption that one reason for oncologists' poor recognition of patient distress and available social support may be that the physicians' interviewing techniques are characterized by directive and closed questions, premature advice and a neglect of psychosocial issues. This discourages patients from disclosing their emotional and psychosocial state. Interviews and group discussions with oncologists showed that they tend to avoid asking their patients about their feelings and the way they cope with illness because they are afraid of provoking strong emotional reactions in their patients (Worden and Weisman, 1980; Maguire et al, 1996; Fallowfield et al, 1998). Oncologists seem to have difficulties in handling their own emotions when confronted with strong emotions of patients or their relatives (Fallowfield and Jenkins, 1999; Maguire, 1999). Caring for cancer patients, and especially for patients who cannot be cured and in whom only palliative treatment is possible, is highly stressful. Cumulative and unresolved stress may lead oncologists to withdraw from communication with patients (Delvaux et al, 1988; Ramirez et al, 1996).
4. Furthermore, class differences between oncologists and patients might hamper effective communication, and decrease awareness of patient distress, perceived social support and need for complementary psychosocial counselling. Communicational barriers between physicians of middle-class origin and lower-class patients have been found in several studies on information provision to the patient (Pendleton and Bochner, 1980; Waitzkin, 1984; DeVries et al, 1998).

We have to acknowledge some methodological shortcomings of this study: (1) We assessed patients' distress with two screening instruments to detect psychosocial problems associated with cancer. Although these questionnaires are validated for use in cancer patients, no assessment of concurrent or former psychiatric disorder by using structured psychiatric interviews was conducted because this was not feasible in the very busy radio-oncological ward. (2) We did not reassess oncologists' and patients' perceptions of distress and social support during the course of radiotherapy. Maybe, physicians might have identified severely distressed patients in need of psychosocial support more accurately when they knew the patient better in the course of treatment. However, such a reassessment is hindered by the organizational procedures of the Radio-Oncology Department: physicians conducting the initial assessment and providing information about treatment are not the same as those responsible for the technical procedures of radiotherapy. (3) Distress or burn-out syndrome in oncologists which might influence their awareness of patients' distress and social support were not evaluated. (4) It was not the aim of this study to examine in detail the characteristics of the

*© 2001 Cancer Research Campaign*

oncologists' interview techniques, but which might help explain why psychological maladjustment to illness was only poorly recognized.

### CONCLUSIONS

This study draws attention to a very real need for methods that help identify severely distressed and poorly supported patients requiring additional supportive counselling. Such methods could comprise (a) screening instruments to detect highly stressed patients and (b) training offered to oncologists to sensitize them to patient distress. There are arguments for both procedures. Passik and colleagues (1998) underline that applying questionnaires for the detection of patient distress and notifying the oncologists of the results may raise the oncologists' awareness of anxiety and depression and facilitate communication between patients and physicians about emotional problems. Maguire (1999), however, raised doubts whether using screening questionnaires is useful and economical because of the low specificity of the instruments available necessitating second phase assessments by mental health professionals of patients scoring above specified threshold values. He rather recommends training health professionals in detecting patient distress.

Communication behaviours that encourage patients to disclose sensitive emotional information include the adoption of a reassuring and empathetic interviewing style, focusing on and clarifying psychosocial aspects, active-listening, using open rather than closed questions, responding to non-verbal cues, as well as empathetic and supportive statements (Putnam and Stiles, 1993; Ford et al, 1996; Maguire, 1999). Such communication skills help to create a trustful relationship between physician and patient which is necessary for addressing emotional problems, especially in patients with denial behaviour. Unfortunately, such communication skills are not sufficiently taught during formal medical education (Fallowfield, 1993; Poulsen, 1998). Particularly, class barriers in doctor–patient communication are seldom addressed (Burnett and Thompson, 1986; VanRyn and Burke, 2000).

However, even empathetic and well-trained oncologists are confronted with numerous clinical tasks, heavy case-load and lack of time for the individual patient in everyday practice. Given that recognizing distressed patients and providing emotional support is not neglected by the physicians, other health care professionals like specially trained oncology nurses might contribute significantly in fulfilling these tasks of good patient care (Maguire et al, 1980; Logan et al, 1999). Recent studies show that oncologists and oncology nurses with training in communication skills more often employ patient-centred interviewing techniques (Razavi et al, 1993; Maguire et al, 1996; Fallowfield et al, 1998), which might help identify more accurately patient distress. There is a need for studies to evaluate the effectiveness of the different procedures on the recognition of patient distress and available social support: the use of screening-instruments, the training of health professionals, and a combination of both measures.

### ACKNOWLEDGEMENTS

This study was supported in part by Janssen & CILAG Pharma, Vienna, and Nutricia, Vienna, Austria.

### REFERENCES

Burnett AC and Thompson DG (1986) Aiding the development of communication skills in medical students. *Med Educ* **20**: 424–431

Delvaux N, Razavi D and Farvaques C (1988) Cancer care – a stress for health professionals. *Soc Sci Med* **27**: 159–166

Derogatis LR, Morrow GR, Fetting J, Penman D, Piasetsky S, Schmale AM, Henrichs M and Carnicke CLM (1983) The prevalence of psychiatric disorders among cancer patients. *JAMA* **249**: 752–757

DeVries A, Söllner W, Steixner E, Auer V, Schiessling G, Sztankay A, Iglseder W and Lukas P (1998) Psychosocial distress and need for supportive counselling in patients during radiotherapy. *Strahlenther Onkol* **174**: 408–414

Fallowfield LJ (1993) Giving sad and bad news. *Lancet* **341**: 476–478

Fallowfield L and Jenkins V (1999) Effective communication skills are the key to good cancer care. *Eur J Cancer* **35**: 1592–1597

Fallowfield LJ, Lipkin M and Hall A (1998) Teaching senior oncologists communication skill: Results from phase I of a comprehensive longitudinal program in the United Kingdom. *J Clin Oncol* **16**: 1961–1968

Ford S, Fallowfield L and Lewis S (1994) Can oncologists detect distress in their out-patients and how satisfied are they with their performance during bad news consultation? *Br J Cancer* **70**: 767–770

Ford S, Fallowfield L and Lewis S (1996) Doctor–patient interactions in oncology. *Soc Sci Med* **42**: 1511–1519

Hardman A, Maguire P and Crowther D (1989) The recognition of psychiatric morbidity on an oncology ward. *J Psychosom Res* **33**: 235–239

Herrmann C (1997) International experience with the Hospital Anxiety and Depression Scale – a review of validation data and clinical results. *J Psychosom Res* **42**: 17–41

Hoffman R, Payne D, Theodoulou M and Massie MJ (1998) Screening for anxiety and depression in women with breast cancer. *Psychosomatics* **39**: 225–229

Hopwood P, Howell A and Maguire P (1991) Screening for psychiatric morbidity in patients with advanced breast cancer: validation of two self-report questionnaires. *Br J Cancer* **64**: 353–356

Iscoe N, Williams JI, Szalai JP and Osoba D (1991) Prediction of psychosocial distress in patients with cancer. In: Osoba D (ed) *Effect of cancer on quality of life*, CRC Press: Boca Raton-Boston-Ann Arbor-London, pp 41–59

Kiss A, Cavalli F, Cull A, Gallmeier WM, Hürny C, Keller M, Kesselring A, Schwarz R, Söllner W, Spiegel D and Stiefel F (1995) Psychosocial/psychotherapeutic interventions in cancer patients: consensus statement. *Supp Care Cancer* **3**: 270–271

Kleining G and Moore H (1968) Soziale Selbsteinstufung (SSE). Ein Instrument zur Messung sozialer Schichten. *Kölner Z Soziol Soz* **20**: 502–552

Lambic C, Nordin K and Sloden PO (1995) Agreement between cancer patients and their physicians in the assessment of patient anxiety at follow-up clinics. *Psycho-Oncology* **4**: 225–236

Logan J, DeGrasse CE, Stacey D, Fiset V and Fawcett L (1999) Oncology nursing education within a supportive care framework: an evidence-based undergraduate course. *Can Oncol Nurs J* **9(2)**: 64–70

Maguire P (1999) Improving communication with cancer patients. *Eur J Cancer* **35**: 2058–2065

Maguire P, Tait A, Brooke M, Thomas C and Sellwood R (1980) Effect of counselling on the psychiatric morbidity associated with mastectomy. *BMJ* **281**: 1454

Maguire P, Booth K, Elliot C and Jones B (1996) Helping health professionals involved in cancer care acquire key interviewing skills – the impact of workshops. *Eur J Cancer* **32A**: 1486–1489

Munro AJ and Potter S (1996) A quantitative approach to the distress caused by symptoms in patients treated with radical radiotherapy. *Br J Cancer* **74**: 640–647

Muthny FA (1989) *Freiburger Fragebogen zur Krankheitsverarbeitung*. Beltz: Weinheim

Newell S, Sanson-Fisher RW, Girgis A and Bonaventura A (1998) How well do medical oncologists' perceptions reflect patients' reported physical and psychological problems? *Cancer* **83**: 1640–1651

Parle M, Jones B and Maguire P (1996) Maladaptive coping and affective disorders among cancer patients. *Psychol Med* **26**: 735–744

Passik SD, Dugan W, McDonald MV, Rosenfeld B, Theobald DE and Edgerton S (1998) Oncologists' recognition of depression in their patients with cancer. *J Clin Oncol* **16**: 1594–1600

Pendleton DA and Bochner S (1980) The communication of medical information in general practice consultations as a function of patients' social class. *Soc Sci Med Med Psychol Med Sociol* **14A**: 669–673

© 2001 Cancer Research Campaign

Poulson J (1998) Bitter pills to swallow. *New Engl J Med* **338**: 1844–1846
Putnam SM and Stiles WB (1993) Verbal exchanges in medical interviews: implications and innovations. *Soc Sci Med* **36**: 1597–1604
Ramirez AJ, Graham J, Richards MA, Cull A and Gregory WM (1996) Mental health of hospital consultants: the effects of stress and satisfaction at work. *Lancet* **347**: 724–728
Razavi D, Delvaux N, Farvacques C and Robaye E (1990) Screening for adjustment disorders and major depressive disorders in cancer in-patients. *Br J Psychiatry* **156**: 79–83
Razavi D, Delvaux N, Marchal S, Bredart A, Farvacques C and Paesmans M (1993) The effects of a 24-hour psychological training program on attitudes, communication skills and occupational stress in oncology: a randomised study. *Eur J Cancer* **29A**: 1858–1863
Rumpold G, Augustin M, Zschocke I, Strittmatter G and Söllner W (2001) The validity of the Hornheide Questionnaire for psychosocial support in skin tumour patients: A survey in an Austrian and a German outpatient population with melanoma. *Psychother Med Psychol* (in press)
Sensky T, Dennehy M, Gilbert A, Bergent R, Newlands E, Rustin G and Thompson C (1989) Physicians' perceptions of anxiety and depression among their outpatients: relationships with patients and doctors' satisfaction with their interviews. *J Roy Coll Physicians* **23**: 33–38
Söllner W, Zingg-Schir M, Rumpold G, Mairinger G and Fritsch P (1998) Need for supportive counselling – the professionals' versus the patients' perspective: A survey in a representative sample of 236 melanoma patients. *Psychother Psychosom* **67**: 94–104
Söllner W, Zschocke I, Zingg-Schir M, Rumpold G, Stein B and Augustin M (1999) Interactive patterns of social support and individual coping strategies in melanoma patients and their correlations with adjustment to illness. *Psychosomatics* **40**: 239–250
Strittmatter G (1997) *Indikation zur Intervention in der Psychoonkologie. Psychosoziale Belastungen und Ermittlung der Betreuungsbedürftigkeit stationärer Hauttumorpatienten*. Waxmann, Internationale Hochschulschriften 228: Münster: New York
VanRyn M and Burke J (2000) The effect of patient race and socio-economic status on physicians' receptions of patients. *Soc Sci Med* **50**: 813–828
Waitzkin H (1984) Doctor–patient communication: clinician implications of social scientific research. *JAMA* **252**: 2441–2446
Worden JW and Weisman AD (1980) Do cancer patients really want counselling? *Gen Hosp Psychiat* **2**: 100–103
Zigmond AS and Snaith RP (1983) Hospital anxiety and depression scale. *Acta Psychiatr Scand* **67**: 361–370

*© 2001 Cancer Research Campaign*
